# TB epidemiology: where are the young women? Know your tuberculosis epidemic, know your response

**DOI:** 10.1186/s12889-018-5362-4

**Published:** 2018-03-27

**Authors:** Rubeshan Perumal, Kogieleum Naidoo, Nesri Padayatchi

**Affiliations:** 1 Centre for the AIDS Programme of Research in South Africa, Nelson R Mandela School of Medicine, College of Health Sciences, University of KwaZulu-Natal, Durban, South Africa; 2 Division of Pulmonology and Critical Care, Department of Internal Medicine, Faculty of Health Sciences, University of Cape Town, Cape Town, South Africa; 3 Medical Research Council-CAPRISA HIV-TB Pathogenesis and Treatment Research Unit, Doris Duke Medical Research Institute, University of KwaZulu-Natal, Durban, South Africa; 4 Centre for the AIDS Programme of Research in South Africa, Doris Duke Medical Research Institute (2nd floor), 719 Umbilo Road, Private Bag X7, Congella, Durban, 4013 South Africa

**Keywords:** Tuberculosis, Human immunodeficiency virus, Epidemiology, Women, Gender

## Abstract

**Background:**

The global predominance of tuberculosis in men has received significant attention. However, epidemiological studies now demonstrate that there is an increased representation of young women with tuberculosis, especially in high HIV burden settings where young women bear a disproportionate burden of HIV. The role of the HIV epidemic, as well as changes in behavioural, biological, and structural risk factors are explored as potential explanations for the increasing burden of tuberculosis in young women.

**Discussion:**

As young women are particularly vulnerable to HIV infection in sub-Saharan Africa, it is unsurprising that the TB epidemic in this setting has become increasingly feminised. This age-sex trend of TB in South Africa is similar to WHO estimates for other countries with a high HIV prevalence where there are more female than male cases notified up to the age of 25 years. The high prevalence of anaemia of chronic disease in young women with HIV is an additional potential reason for their increased TB risk. The widespread use of injectable medroxyprogesterone acetate contraception, which has been shown to possess selective glucocorticoid effect and oestrogen suppression, in young women may be an important emerging biological risk factor for tuberculosis in young women. Behavioural factors such as alcohol use and tobacco smoking patterns are further factors which may be responsible for the narrowing of the sex gap in TB epidemiology. In comparison to the significantly higher alcohol consumption rates in men globally, there is a narrowing gap in alcohol consumption between the sexes in South Africa with alarming rates of alcohol abuse in young women. There is a similar narrowing of the tobacco smoking gap between the sexes in South Africa, with increasing smoking prevalence in young women.

**Conclusion:**

With nearly 70% of all TB patients being co-infected with HIV in our setting, it is not surprising that the age and sex distribution of TB is increasingly resembling the distribution of HIV in this region of dual hyperendemicity. New TB service design must begin to reflect the presence of young women as a significant group burdened by the disease.

## Background

With a global incidence of 13.4 million new cases in 2015, tuberculosis remains a public health priority which demands our ongoing efforts toward its successful control [1]. In South Africa, tuberculosis is now the leading cause of death, fuelled by a burgeoning HIV epidemic [2]. Unlike HIV programmes, TB control efforts have not enjoyed a similar focus on key populations, and a detailed understanding of local disease epidemiology has been substituted for a uniform public health strategy. However, there has been renewed interest in deepening our understanding of the disease epidemiology especially with regard to sex differences. In contrast to the significant attention that has been given to the global predominance of tuberculosis in men, there has been a paucity of focus on the burden of disease in young women, particularly in high TB/HIV burden settings. In this article we argue that there is an increasing burden of TB in young women in high HIV burden settings resulting from biological, behavioural and structural risk factors which require further evaluation. The opportunity to redesign TB services so that it appropriately responds the needs of young women is also explored.

## Discussion

The excess of notified cases among men has been explained by increased transmission between men, the increased prevalence of proximate risk factors for TB such as smoking and alcohol use, specific immunological vulnerabilities in men, barriers faced by women in seeking care and presenting for diagnosis, and most recently, biological predispositions in men related to iron metabolism [3–6]. Although sex disaggregation of global and local tuberculosis data is an important starting point for unpacking TB epidemiology, age-specific sex ratios may offer more useful insights into local disease epidemiology and should serve as the basis for health system planning. While the overall male: female(M:F) TB prevalence ratio was 2.21 for bacteriologically positive tuberculosis in low and middle-income countries, the age-specific sex ratios ranged from 1.28 among individuals aged 15–24 years to 3.18 among individuals aged 45–54 years in a recent meta-analysis [7]. Moreover, M: F TB prevalence ratios were lower, and demonstrated a female predominance, in settings of high HIV prevalence (0.67, 95% CI 0.49–0.90; 54 surveys). The specific exclusion of studies in HIV sub-populations in the meta-analysis, results in a failure to reflect the epidemiological nuances in high TB/HIV burden settings. Importantly, seven out of the top 20 high TB/HIV burden countries (six out of the seven being African countries) were not represented in the meta-analysis, likely as a result of the lack of high quality epidemiological studies in these parts of the world. As young women are particularly vulnerable to HIV infection in sub-Saharan Africa, it is unsurprising that the TB epidemic in this setting has become increasingly feminised.

There have been several studies evaluating the factors which may contribute to the under-representation of women in epidemiological assessments of tuberculosis. The study of only bacteriologically proven tuberculosis, as is the case with the meta-analysis by Horton et al., systematically under-represents women who are more likely to be sputum non-producers and are more likely to experience smear-negative tuberculosis than their male counterparts [8, 9]. This is of heightened importance in studies conducted prior to the programmatic rollout of Xpert MTB/RIF, when sputum microscopy served as the primary microbiological basis for the diagnosis of tuberculosis in many low and middle income countries. The exclusion of studies employing active case finding or enhanced case finding may have also contributed to the exclusion of a disproportionately greater number of female cases of TB as active case finding has been demonstrated to identify a greater number of young women with HIV-associated TB [10]. An additional reason for the under-representation of women with TB in epidemiological studies is the greater number of missed tuberculosis cases in women despite their contact with health systems. TB symptom screening – a key component of intensified case finding - has also been shown to be of limited value in HIV-positive women, especially pregnant women, and missed 72% of cases of asymptomatic prevalent TB [11].

Heavy alcohol use has a complex and detrimental effect on systemic and lung immunity predisposing alcohol abusers to the acquisition of tuberculosis. The global pattern of significantly higher rates of drinking among men has been suggested as one of the factors resulting in a male predominance of TB. It is, however, alarming that while men have a higher overall prevalence of alcohol use in South Africa, this consumption gap is narrowing [12]. Although the per capita alcohol consumption in sub-Saharan Africa are similar to global averages, the region also has one of the highest rates of abstinence from alcohol, with very high levels of consumption amongst those who do drink [13]. In the Western Cape province of South Africa, where some of the highest incidence rates of TB occur, 34% of urban and 46–51% of rural women drink during pregnancy, effectively closing the drinking gap between these young women and their male counterparts [14]. Differential patterns of smoking between sexes have been postulated to explain up to one third of the sex differences seen in global TB epidemiology [15]. While smoking is still twice as common in South African men than women, the only group with an increasing smoking prevalence are young, educated (>grade 12) women in the setting of urbanisation [16]. These patterns of alcohol and smoking use may well be contributing to the narrowing of the sex gap in local TB epidemiology.

Studies of hormonal mechanisms and TB suggest the protective effect of oestrogen and the potentially deleterious effect of medroxyprogesterone acetate on TB acquisition and disease control. A murine model with comparable serum concentrations as injectable medroxyprogesterone acetate contraception demonstrated significant glucocorticoid effect, and significantly increased risk of TB acquisition and reduced control of TB disease [17]. A similarly increased immunological vulnerability to TB was demonstrated in an in vitro human study on the effect of progesterone contraception on immunological correlates of TB risk [18]. The widespread use of the progesterone contraceptive Depo-Provera in South Africa may be contributing to a loss of oestrogenic protection and an increased glucocorticoid effect in South African women, conferring an increased risk of TB [17, 19]. Importantly almost half of all women of child-bearing age are on contraception in South Africa, and the use of injectable progesterone contraception is now the most commonly used method of contraception, especially amongst young women [19]. The popularity of this form of injectable contraception has an appeal to young women the world over because, apart from its efficacy, it requires only four clinic visits per year and obviates the need for a daily oral contraceptive. Androgen deprivation by castration in male mice leads to an increase in T-lymphocytes in lymph nodes and increased proliferation of these cells when activated by antigenic stimulation [20]. Further, epidemiological evidence of oestrogenic protection is revealed by studies which demonstrate significantly poorer TB outcomes in intact men compared to castrated men, and increased TB mortality in women who underwent oophorectomy [21, 22]. Key postulates are that oestrogen supports the activation of macrophages, a key component of the immunological control of TB, and that oestrogen plays an important role in amplifying interferon-gamma secretion by activated natural killer T-cells [23]. The effect of ovarian follicular stagnation and resultant hypooestrogenism by long term medroxyprogesterone acetate exposure has been demonstrated, and implications for oestrogen sensitive tissues such as components of the immune system require further evaluation [24, 25]. The protective role of oestrogen, mediated through its synergism with interferon-gamma production, against pathogens such as *Coxiella, Leishmania,* and other mycobacteria have been previously reported [26–28]. Serum oestrogen levels in long term injectable progesterone contraceptive users may be as low as the post-menopausal range, and may be plausibly linked to the loss of the protective benefit of oestrogen on TB acquisition and disease control in these, predominantly young, women. These sex-hormone effects on TB risk are supported by the highest M:F ratios during the reproductive years in women globally, while the sex ratio approximates 1:1 in the pre-pubescent age group [4, 29].

**Fig. 1:**
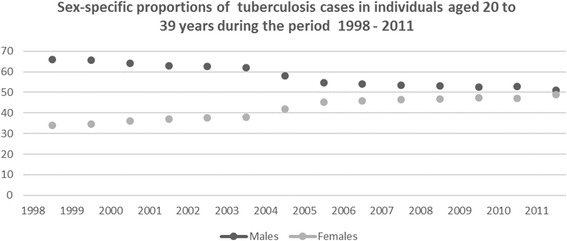
Proportions of men and women with sputum positive tuberculosis aged 20 to 39 years at an urban facility in Durban, South Africa during the period 1998 to 2011. Unpublished review of programmatic data from an urban TB clinic in Durban, South Africa (Ethical approval BFC031/08)

Even in the face of a global predominance of TB in men, it cannot be ignored that TB/HIV co-infected women experience mortality rates that are 20% higher than TB/HIV co-infected men [30]. The global and local epidemiology of TB has shifted over the decades with the advent of HIV from being a disease with historically increased prevalence in men, and in those at the extremes of age, to one of increasing prevalence in women [1]. This shifting demographic feature has not been accounted for in health system planning activities, with little emphasis on making tuberculosis facilities more women’s health friendly. This is particularly important in sub-Saharan Africa where the local tuberculosis epidemiology has been shaped by the intervening HIV epidemic, and the disproportionate burden of HIV in young women. HIV is now widely recognised as the most potent risk factor for the development of tuberculosis disease, with a 21–34 times higher risk of tuberculosis in people living with HIV [1]. We previously reported a female predominance of tuberculosis in adults under 30 years of age in Durban, South Africa, an epidemiological effect which was greater in the HIV positive subset of patients [31]. The inference is that the well documented higher prevalence of HIV in young women compared to men is driving the increased prevalence of tuberculosis in this group, resulting in the feminisation of the TB epidemic in young patients in this setting. A previous study in this setting demonstrated a significantly higher burden of TB disease among women in the 20 to 29 years age group, in keeping with predictions by mathematical models on the impact of HIV on TB in areas of dual hyperendemicity [32]. In addition, patients under the age of 30 years constitute approximately a third of all patients with tuberculosis locally. While the overall global male predominance of tuberculosis requires greater attention, it is essential that we do not neglect the growing number of young women with tuberculosis, especially given their unique socio-economic vulnerability in low and middle-income countries. Unpublished programmatic data from an urban tuberculosis facility in Durban, South Africa (Ethical approval from the University of KwaZulu-Natal Biomedical Research Ethics Committee, BFC031/08) demonstrates that the proportion of women with tuberculosis aged 20–39 years has steadily increased, in keeping with the increase in HIV prevalence among young women (Fig. 1).

This age-sex trend of TB in South Africa is similar to WHO estimates for other African countries with a high HIV prevalence where there are more female than male cases notified up to the age of 25 years [33]. Importantly, in low income countries TB kills more women than all causes of maternal mortality combined. Young women have up to 34% higher risk of progression to TB disease than men up to the age of 30 years attributed to reduced immunity associated with the stresses of pregnancy [34]. In addition, young women have lower cumulative TB infections in adolescence, inferring that new post-adolescence infection might be significantly greater amongst women. New infection with TB carries a greater likelihood to progress to TB disease, and to do so more rapidly.

Iron is a critical element for the survival and proliferation of *Mycobacterium tuberculosis* [35]. More than 80% of patients with HIV associated TB are anaemic in South Africa, with over 95% of these cases resulting from anaemia of chronic disease [36]. This form of anaemia is characterised by an elevation of the iron-gating hormone hepcidin, which traps iron within the reticuloendothelial system and macrophages [37]. Macrophage iron loading may confer an increased risk for TB acquisition and progression to disease by providing an essential substrate for the organism’s survival and growth. The disproportionate burden of iron-deficiency anaemia in women of reproductive age, due largely to menstrual losses, has been cited as a factor conferring protection from TB in women and potentially explains the male predominance of TB globally in these age groups. However, in high HIV burden settings, the prevalence of anaemia of chronic disease is high with a significantly increased burden of this type of anaemia in women compared to men [38]. It is possible that the high burden of anaemia of chronic disease in women with HIV reduces the protection from TB that accrues from iron-deficiency, and moreover that macrophage iron loading in these women increases their risk of TB. Importantly, anaemia may ensue early after HIV acquisition, and may represent the initial effects of hepcidin elevation during the period of sustained systemic inflammation following acute HIV infection [39]. These factors support the hypothesis that iron loading of macrophages in young women, who bear the greatest burden of incident HIV in sub-Saharan Africa, are at an increased risk for TB disease, and may explain the lower M:F TB ratios in high HIV burdened settings in sub-Saharan Africa.

A growing recognition of the gradually changing face of TB will allow for the exploitation of the opportunities presented by this evolving epidemiology. The broadening of services may serve to appropriately orientate existing TB services with regards to the changing demographic profile of patients, to augment women’s health services, to respond to the need for comprehensive primary health care services, and most importantly, to acknowledge the presence of multiple pathologies in an individual patient that cannot adequately be addressed by a vertical programme design. This might include access to family planning, pap smear evaluations, and targeted interventions for young women such as microbicide gels in future.

## Conclusions

The problem of TB/HIV co-infection is not a homogenous entity, but rather a composite of many complex sub-epidemics. This is largely due to the various drivers of HIV and TB which have differential effects on various strata of the population, most notably sub-populations by age and sex. A key component of our response to tuberculosis must be our attention to the complexity of our local epidemics. With nearly 70% of all TB patients being co-infected with HIV in our setting, it is not surprising that the age and sex distribution of TB is increasingly resembling the distribution of HIV in this region of dual hyperendemicity. Whether this is due to behavioural, biological or structural reasons is still uncertain, but the greater representation of women at TB facilities in high HIV burden settings has multiple programmatic implications. A more comprehensive women-friendly service may lead to greater public access, more equitable access for all patients, a more convenient and satisfying service, and better health overall. New TB service design must begin to reflect the presence of young women as a significant group burdened by the disease.

## Abbreviations

HIV: Human immunodeficiency virus
TB: tuberculosis
